# Diffusion tensor-based fiber tracking of the male urethral sphincter complex in patients undergoing radical prostatectomy: a feasibility study

**DOI:** 10.1186/s13244-020-00927-x

**Published:** 2020-11-27

**Authors:** Ana S. C. Verde, Joao Santinha, Eunice Carrasquinha, Nuno Loucao, Ana Gaivao, Jorge Fonseca, Celso Matos, Nikolaos Papanikolaou

**Affiliations:** 1 Head of Computational Clinical Imaging Group, Centre for the Unknown, Champalimaud Foundation, Av. Brasilia, 1400-038 Lisbon, Portugal; 2 Philips Healthcare, Lisbon, Portugal; 3 Radiology Department, Champalimaud Foundation, Lisbon, Portugal; 4 Urology Unit, Champalimaud Foundation, Lisbon, Portugal; 5 Instituto de Ciências Biomédicas Abel Salazar, Universidade Do Porto, Porto, Portugal

**Keywords:** Diffusion tensor imaging, Fiber tracking, Prostatic neoplasms, Urethral sphincters, Urinary incontinence

## Abstract

**Objectives:**

To study the diffusion tensor-based fiber tracking feasibility to access the male urethral sphincter complex of patients with prostate cancer undergoing Retzius-sparing robot-assisted laparoscopic radical prostatectomy (RS-RARP).

**Methods:**

Twenty-eight patients (median age of 64.5 years old) underwent 3 T multiparametric-MRI of the prostate, including an additional echo-planar diffusion tensor imaging (DTI) sequence, using 15 diffusion-encoding directions and a *b *value = 600 s/mm^2^. Acquisition parameters, together with patient motion and eddy currents corrections, were evaluated. The proximal and distal sphincters, and membranous urethra were reconstructed using the deterministic fiber assignment by continuous tracking (FACT) algorithm, optimizing fiber tracking parameters. Tract length and density, fractional anisotropy (FA), axial diffusivity (AD), mean diffusivity (MD), and radial diffusivity (RD) were computed. Regional differences between structures were accessed by ANOVA, or nonparametric Kruskal–Wallis test, and post-hoc tests were employed, respectively, TukeyHSD or Dunn’s.

**Results:**

The structures of the male urethral sphincter complex were clearly depicted by fiber tractography using optimized acquisition and fiber tracking parameters. The use of eddy currents and subject motion corrections did not yield statistically significant differences on the reported DTI metrics. Regional differences were found between all structures studied among patients, suggesting a quantitative differentiation on the structures based on DTI metrics.

**Conclusions:**

The current study demonstrates the technical feasibility of the proposed methodology, to study in a preoperative setting the male urethral sphincter complex of prostate cancer patients candidates for surgical treatment. These findings may play a role on a more accurate prediction of the RS-RARP post-surgical urinary continence recovery rate.

## Key points

- Urethral sphincter complex DTI is feasible at 3 T for prostate cancer patients.
- Subject motion and eddy currents corrections did not significantly influenced DTI metrics.
- DTI derived metrics differentiate the structures of the patients’ urethral sphincter complex.
- Study findings are a start point to predict post-surgical continence recovery rate.

## Introduction

The male urethral sphincter complex is responsible for the maintenance of continence in rest and stress conditions and is comprised of two structural components, the proximal lissosphincter and the distal rhabdosphincter [1]. Whereas the former is composed of smooth muscle and forms a complete ring around the urethra, the latter is made of skeletal muscle and has a shape of a horseshoe. Patients that undergo radical prostatectomy for treating prostate cancer may become urinary incontinent due to damage of their urethral sphincter complex or its fascial framework. A novel robotic surgical technique for radical prostatectomy, the Retzius-Sparing Robot-Assisted Laparoscopic Radical Prostatectomy (RS-RARP) was recently proposed by Galfano in order to preserve most of the structures related to urinary incontinence and therefore to reduce postoperative incontinence [2]. RS-RARP spares the retropubic space by passing through the Douglas pouch and thus avoids bladder mobilization and preserves the endopelvic fascia, the Santorini’s plexus and the puboprostatic ligaments; structures that are damaged in the conventional retropubic robotic radical prostatectomy.

Despite the latter, post-surgical urinary incontinence is still a common and incompletely understood long-term limiting side effect that severely impacts a patients’ quality of life [3]. The knowledge of the microarchitecture of the male urethral sphincter complex can open a window into post-surgical incontinence preclusion. To study the male urethral complex, apart from the pelvis dissection in cadavers [1], in-vivo methods such as elastography using ultrasound [4], electromyography [5], micro-computed tomography [6], evacuation proctography [7], transurethral ultrasound [8] or MRI have been employed. Moreover, DTI is an MRI-based technique that probes water diffusion in multiple directions allowing a 3D visualization of the urethral sphincter complex fibers in terms of size, location, directionality, and integrity. After a wide application of DTI on the central nervous system, the advent of EPI that minimizes motion-related artifacts by acquiring multiple echoes [9] has allowed to extend the use of DTI to other organs including those of the abdominal and pelvic regions (e.g., kidney, liver, pancreas, uterus, and prostate) [10]. In particular, muscle fibers and nerves present a highly ordered microstructure that makes them, in theory, ideally suited to be analyzed by DTI with fiber tracking [11]. DTI of the prostate and adjacent structures has been focused on differentiating between benign and malignant prostate tissue [12], investigating age-related differences of the prostate [13], studying the periprostatic neurovascular fibers and its relation with post-surgical erectile dysfunction [14], and more recently, exploring the male urethral sphincter complex microstructure in eleven healthy young subjects [15].

The aim of the present study was to visualize, map, and extract relevant DTI metrics in a larger dataset of male patients with prostate cancer that underwent RS-RARP, establishing a method for acquiring and processing this type of data. We intended to optimize acquisition parameters considering scan time and in-plane resolution, to select fiber tracking parameters for a deterministic tractography algorithm and to evaluate the need for eddy currents and patient motion correction. So, this study’s main purpose was to prove the technical feasibility of a 3 T MR-DTI sequence and to establish normative DTI metrics that could differentiate between the structures of the male urethral sphincter complex of prospective RS-RARP patients.

## Materials and methods

### Patient population

**Fig. 1:**
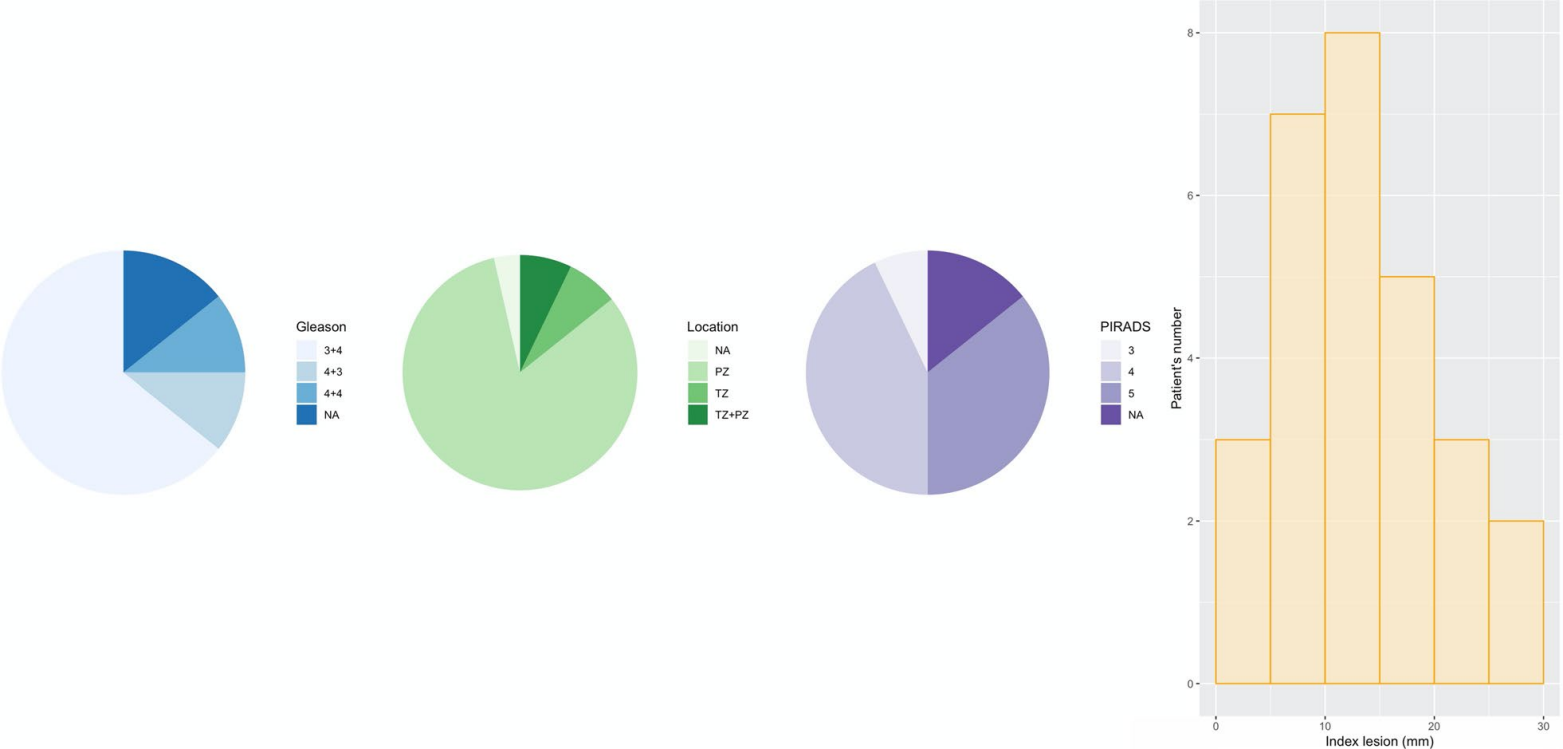
Descriptive statistics on the tumor characteristics, from left to right: pie charts representing the number of patients within each category of Gleason score, tumor location, and PI-RADS score, where “NA” means that information was not available; histogram showing the tumor index lesion size (maximum length in mm) distribution for the same patients

Thirty patients diagnosed with localized or locally advanced prostate cancer have completed a prostatic multiparametric MRI before being submitted to RS-RARP. No patient was excluded due to typical MR exclusion criteria, such as claustrophobia or the presence of implanted medical devices. However, two of the first three patients used for acquisition parameters optimization were excluded from the subsequent analysis for having different acquisition parameters. In the end, twenty-eight patients with median age 64.5 years old (ranging from 48 to 72 years old) were used for the processing and quantitative image analysis. Numeric variables age, body mass index (BMI), preoperative prostate-specific antigen (PSA) levels in ng/mL, and prostate volume (PV) in cm^3^ were collected (Table 1). Additional variables describing the tumor characteristics are graphically represented on Fig. 1. These comprise three categorical variables and one numeric, respectively, biopsy Gleason score, tumor location, PI-RADS v2 (2015) score, and index lesion size. This prospective study was approved by the local ethics committee and all patients involved gave written informed consent in this prospective study.

**Table 1:** Descriptive statistics on the patient’s dataset (28 patients)

Variable name	Median	IQR [min-max] (*N*)
Patient age (years old)	64.5	7 [48–72] (28)
BMI (kg/m^2^)	27.1	3.57 [21.7–33.2] (28)
Preoperative PSA (ng/mL)	7.0	4.55 [2.0–15.1] (24)
PV (cm^3^)	43	20 [25–76] (28)

Each numeric variable was presented as Median; IQR [minimum–maximum] (*N*), where IQR stands for interquartile range and *N* for the number of patients for which that variable information was available

### Data acquisition

**Fig. 2:**
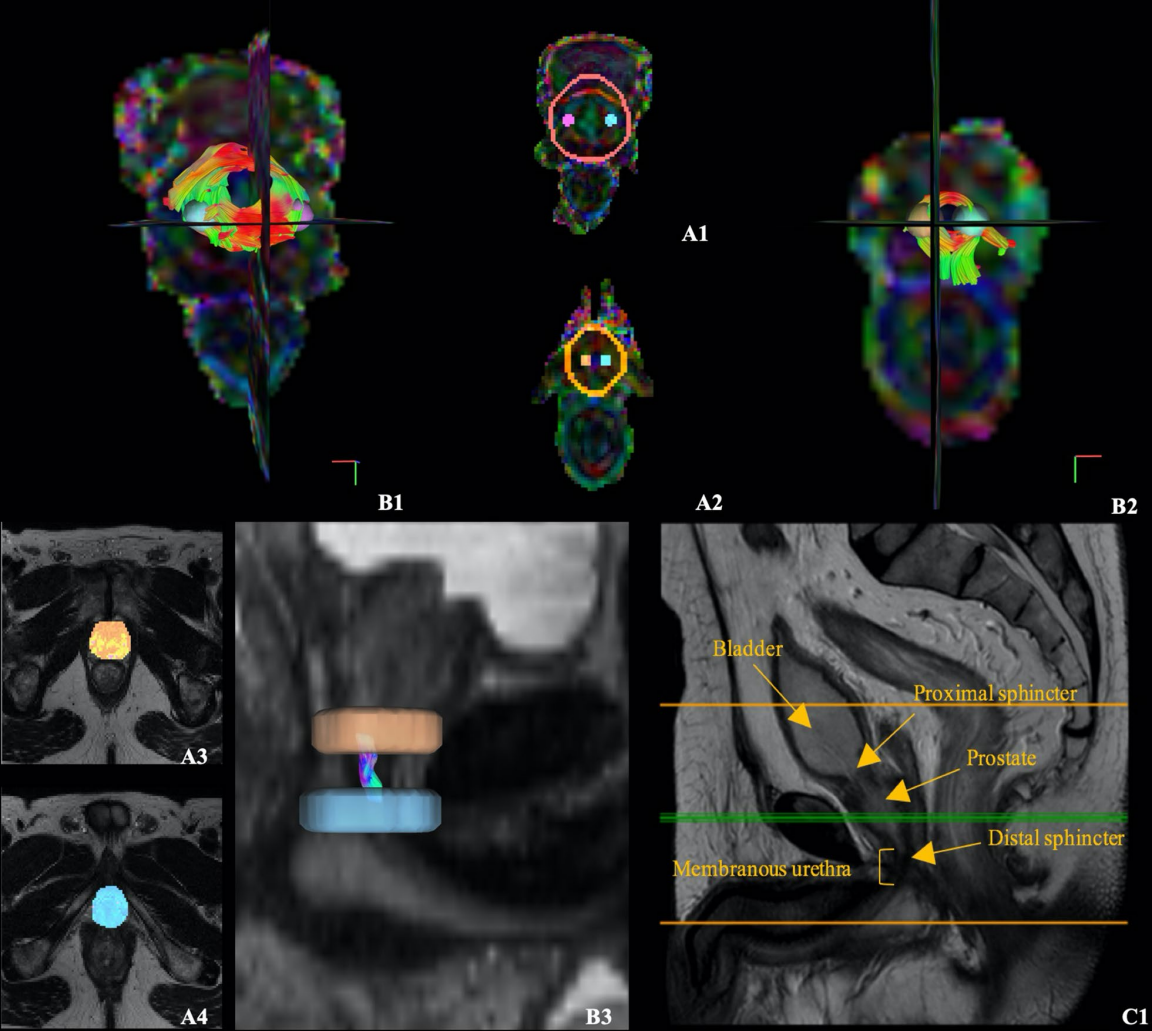
Axial views obtained from DSI Studio showing the region of interest (ROI) placement on the color-coded FA maps for the proximal sphincter (**A1**) and distal sphincter (**A2**), and on the T2-weighted image for the apex (**A3**) and bulb (**A4**) to track the membranous urethra. Fiber tracking results derived from the DTI images of an arbitrarily chosen study patient are shown for the proximal sphincter (**B1**), distal sphincter (**B2**), and membranous urethra (**B3**), color-coded based on the direction of the fibers—red for left–right (x), green for anterior–posterior (y), and blue for superior-inferior (z) directions. MRI T2-weighted image of a patient’s mid-sagittal slice labelled with the relevant anatomy, where the orange lines define the proximo-distal extent imaged in the DTI acquisition (**C1**)

Patients were scanned at a 3 T MRI scanner (Ingenia, Philips Healthcare, The Netherlands), equipped with a 32-channel phased-array abdominal coil, parallel imaging, and multi transmission radio-frequency capabilities. All participants were asked to fast for 4 h before the examination and to apply a microlax to minimize intestinal peristalsis that causes artifacts, although no empty bladder requirements were imposed. Apart from the standard clinical multiparametric-MRI protocol—including a T2-weighted sequence with the following parameters: echo time = 125 ms, repetition time = 4900 ms, matrix size = 288 × 230 × 30 mm^3^, field of view = 200 × 200 × 90 mm^3^, number of averages = 2, in-plane resolution = 0.31 × 0.31 mm^2^, slice thickness = 3 mm, slice number = 30, gap = 0.3 mm -, it was also implemented a DTI sequence for 3D visualization of the male urethral sphincter complex. This sequence was acquired in the Anterior–Posterior phase encoding direction by means of single-shot diffusion-weighted echo-planar imaging (DW-EPI) with parallel SENSE (sensitivity encoding) technique, using a reduction factor of 2. Acquisition parameters included 15 diffusion-encoding directions, b-value of 0 (b0) and 600 s/mm^2^, echo time = 68 ms, repetition time = 6600 ms, matrix size = 68 × 65 × 40 mm^3^, field of view = 200 × 200 × 120 mm^3^, number of averages = 1, in-plane resolution = 1.39 × 1.39 mm^2^, slice thickness = 3 mm, slice number = 40, gap = 0.3 mm, representing a sequence time of four minutes. Acquisition coverage, including the whole prostate gland and penile bulb, can be depicted in Fig. 2—C1. Two of the first patients were scanned with a DTI protocol incorporating 32 diffusion encoding directions and b-value of 0 (b0) and 800 s/mm^2^, for comparison purposes. For a subset of 17 patients, each image volume was co-registered to the baseline image (without diffusion weighting) to correct for patient motion. Additionally, for five of these patients, a DTI sequence was scanned with a b-value of 0 s/mm^2^ (b0) in the Posterior-Anterior (PA) phase-encoding direction to further correct for susceptibility induced distortions.

### Data processing and fiber tractography

**Fig. 3:**
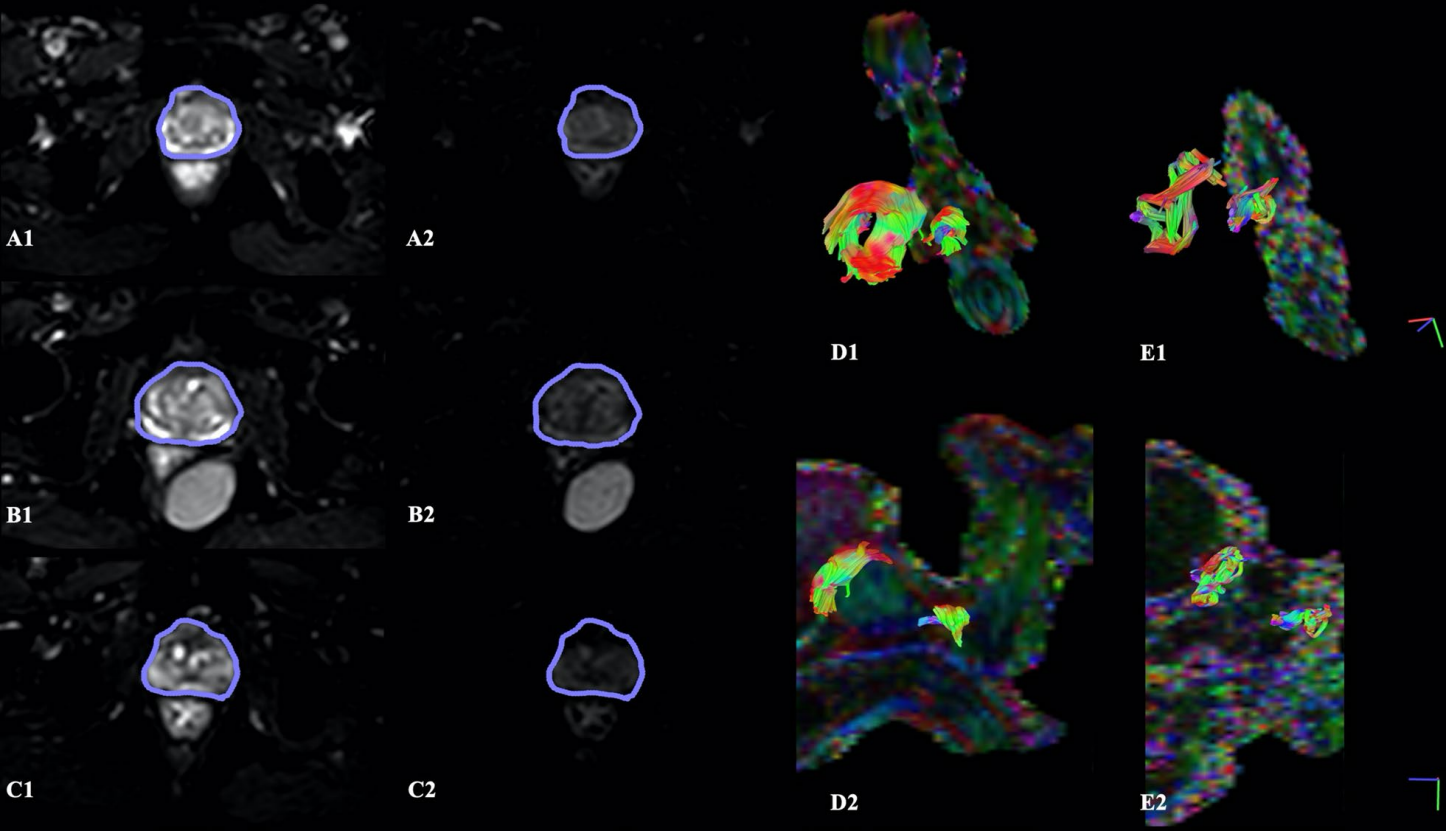
On the left, three patients with diffusion-weighted images at the level of the prostate with its contour manually traced on the baseline *b* = 0 s/mm^2^ image (left column), overlaid on the *b* = 600 s/mm^2^ at the corresponding location (right column), to verify that eddy currents did not significantly caused distortions on the prostate. On the right, comparison of fiber tracking results for the sphincters and membranous urethra using two different combinations of acquisition parameters: (1) *b *value of 600, 15 directions, 40 acquired slices (**D1** and **D2**, from two different views); (2) *b *value of 800, 32 directions, 25 acquired slices (**E1** and **E2**)

To evaluate if the eddy currents induced-distortions were significant, the prostate contour was manually traced by the first author on the b0 image and overlaid on the DW image with a higher b-value and verified by a radiologist with more than 10 years of clinical experience in prostate imaging (Fig. 3—A1 to C2). Two different approaches for eddy currents correction were used on data from two subsets of patients, respectively, the 17 patients used for motion correction and five of these patients with the acquisition on an opposite phase-encoding direction. The first was the motion/eddy currents correction option on DSI Studio [16], based on an affine transformation between the diffusion b0 and each diffusion-weighted volume, using image correction as the cost function. The second was *eddy* from FSL after calculating the susceptibility-induced off-resonance field using TOPUP that follows the method described in [17] as implemented in FSL [18].

Tensor estimation and fiber tractography were performed using DSI Studio with the deterministic fiber assignment by continuous tracking (FACT) algorithm [16] for fiber propagation. This algorithm is based on computing a 3-D trajectory in continuous space starting at a user-defined region of interest (ROI). For the proximal and distal sphincters, three ROIs were positioned on the color-coded fractional anisotropy (FA) maps. Two spherical AND gates were placed symmetrically on both sides of the ring and one terminative surrounding cylindrical-shaped ROI to restrain the tracking of fibers outside the structure (Fig. 2A1 and A2). For the membranous urethra, the T2-weighted volumes (moving image) were registered to the b0 volumes of the original DTI (fixed image) using rigid followed by affine transformations, implemented using the SimpleElastix library [19] in Python. Using the T2-weighted aligned image as anatomical reference, two ROIs were placed at the level of the prostate apex and penile bulb (Fig. 2—A3 and A4). The ROI placement was validated by coauthors J.F and A.G, medical doctors with 30 and 19 years of experience, respectively.

**Fig. 4:**
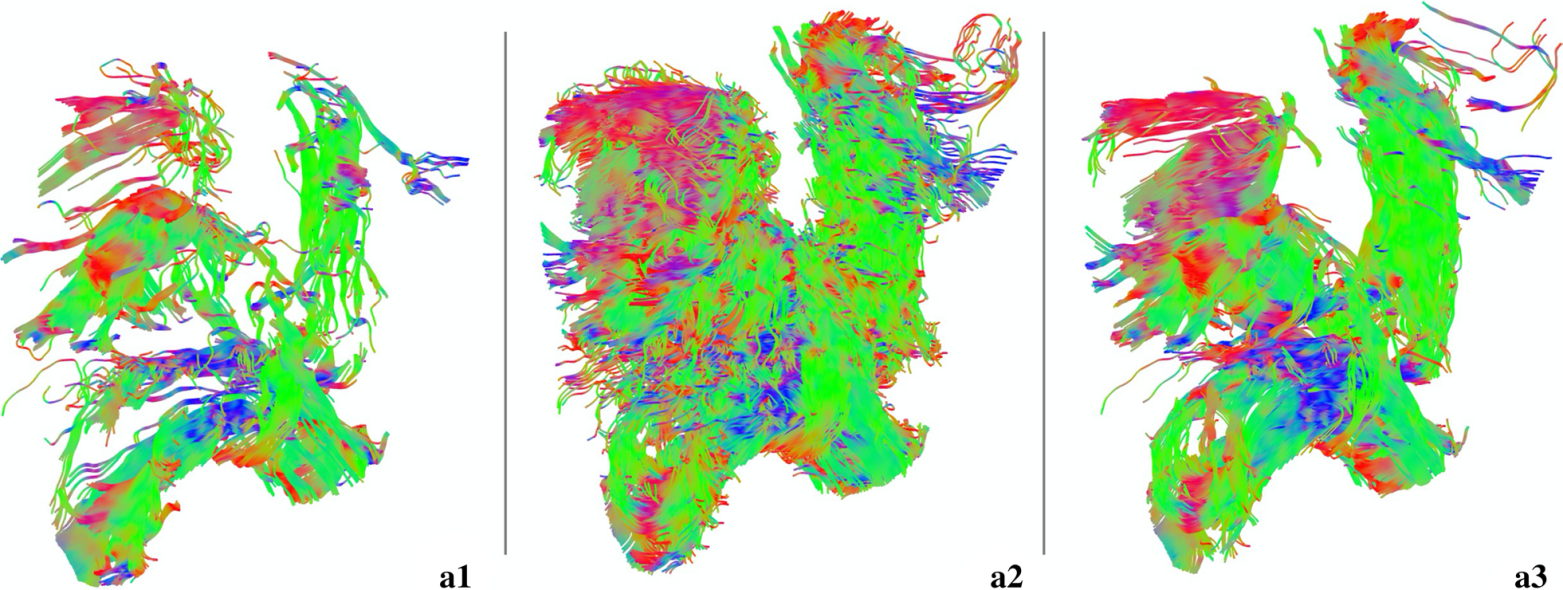
Comparison of different thresholds for DTI fiber tractography in the prostate and surrounding areas of a 61-year-old prostate cancer patient. Fiber tractography results generated using the following combinations of FA thresholds and angular thresholds: 0.25 with 75º (**a1**), 0.15 with 75º (**a2**), and 0.15 with 50º (**a3**)

**Fig. 5:**
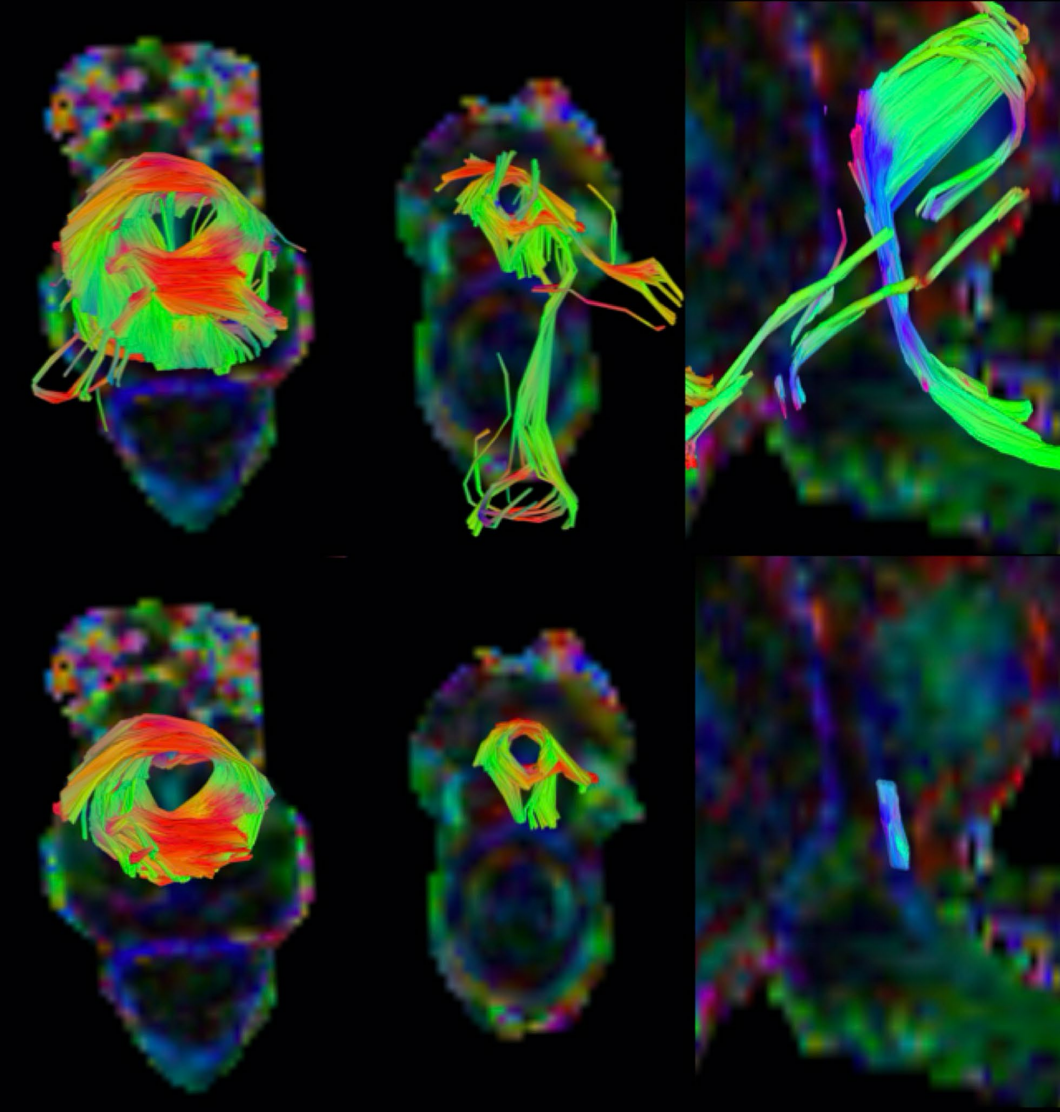
A representative proximal and distal sphincters and membranous urethra before and after the deletion of spurious fibers on DSI Studio, respectively, from top to bottom

**Fig. 6:**
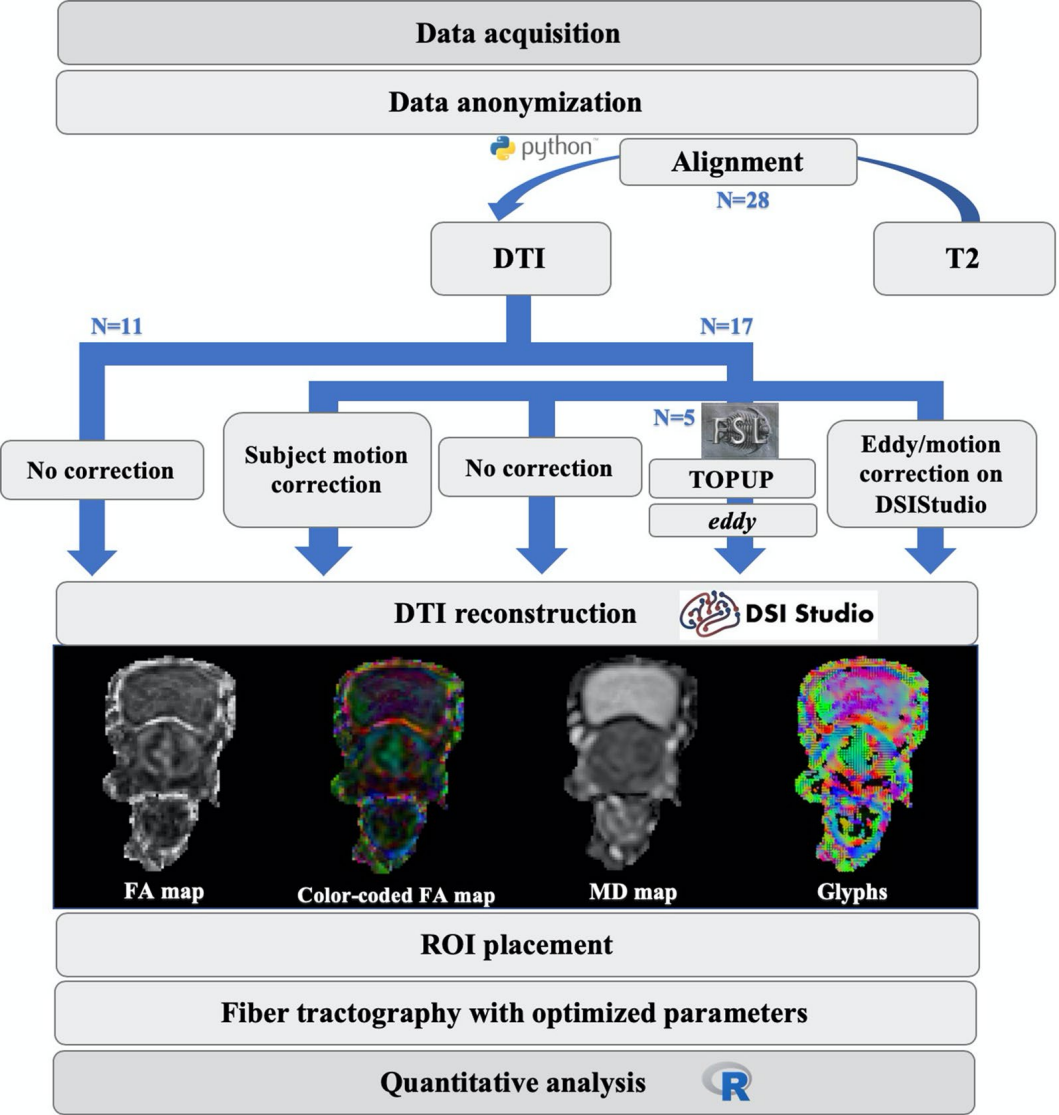
Schematic representation on the study DTI processing and fiber tractography pipeline

To determine optimal FA and angular thresholds, seeds were placed on the prostate and surrounding areas and a whole region fiber tractography was constructed while varying the thresholds manually (Fig. 4). Based on these scenarios, it was decided that fiber tracking terminated if one of these conditions were met: (1) tract length shorter than 50 or longer than 200 mm; (2) FA threshold lower than 0.15; (3) angular threshold higher than 45º for the membranous urethra or higher than 75º for the sphincters. Additionally, a trilinear direction interpolation was used, and fiber trajectories were smoothed by averaging the propagation direction with 9% of the previous direction. A step size of 0.69 mm was utilized and a total of 30,000,000 seeds were placed. After tracking, spurious tracts were discarded manually (an arbitrarily chosen example for each one of the structures can be observed on Fig. 5), and, for the membranous urethra, tracts above the apex or below the bulb were eliminated. A schematic processing pipeline can be depicted in Fig. 6.

### Quantitative analysis

For each structure of every patient, seven DTI metrics were automatically calculated, the tract length (in mm) number and volume (in cm^3^), fractional anisotropy (FA), axial diffusivity (AD), mean diffusivity (MD), and radial diffusivity (RD) were extracted. FA is dimensionless, whereas AD, MD, and RD are in 10^–3^ mm^2^/s. Tract density was calculated by dividing the number of tracts by the volume occupied. Regional differences, meaning intra-subject DTI metrics differences between the proximal and distal sphincters and membranous urethra, were accessed by a one-way ANOVA (or the nonparametric Kruskal–Wallis in the cases were the parametric assumptions were violated). Additionally, the same statistical tests were used to compare DTI metrics using the two eddy currents correction methods and without correction. The comparison of DTI metrics with and without patient motion correction was done using a student’s t-test (or the nonparametric Mann–Whitney). Tukey or Dunn post-hoc tests followed ANOVA or Kruskal–Wallis, respectively. Statistical testing was conducted in Software R, v.3.5.0 [20], using a level of significance, α, was set to 5%.

## Results

### Optimization of acquisition and fiber tracking parameters

Acquisition parameters for DTI of the male urethral sphincter complex were chosen based on the assessment of image resolution and scan time. Voxel size and scan time were intended to be reduced, the former in order to improve the resolution of the fibers and the latter in order not to prolong much the total sequence time, making it feasible for clinical implementation. Since fiber tracking results with 15 directions and b-value of 600 created better fiber tracking results than using 32 directions and b-value of 800 (Fig. 3—D1 to E2), and it represented a shorter scan time and a better resolution (Table 2), those were the established parameters. That way, one can acquire more slices by a shorter scan time that was only possible with the prior parameters by a large decrease in the number of slices, that will preclude the tracking of the complete distal sphincter. Additionally, optimal fiber tracking parameters were empirically derived. For defining the FA and angular thresholds, a whole-prostate fiber tractography was constructed by placing seed-points over a patient's entire organ and varying the thresholds. The results have determined that decreasing the FA threshold to 0.15 and increasing the angular threshold to 75º (Fig. 4—A2), it results in an observed higher density of tracts that better outlines the anatomy of the involved structures. On the opposite side, the other two configurations show a reduction in the number of tracts that appear to be more dispersed (Fig. 4—A1) and less sensitive to tracts with greater curvature (Fig. 4—A3). Although not suitable for the sphincters, a lower angular threshold of 45º demonstrated to accurately depict a straight structure like the membranous urethra.

**Table 2:** Optimization of DTI acquisition parameters for the first three study patients

Case	*b* Value (s/mm	Number of directions	Number of slices	In-plane resolution (mm^2^)	Sequence duration
001’	800	32	25	1.56 × 1.56	7 min 46 s
002’	800	32	20	1.56 × 1.56	6 min 26 s
001	600	15	40	1.39 × 1.39	4 min 9 s

### Effect of patient motion and eddy currents corrections

Although the advent of EPI imaging has extended the use of DTI to other structures beyond the brain, with a reduction on scan time, it is also more sensitive to some artifacts like eddy currents distortions. The shape of the prostate contour on the b = 600 s/mm^2^ image superimposed the shape of the prostate on the diffusion baseline image, for three arbitrarily chosen patients (Fig. 3—A1 to C2). This suggests that the prostate was not significantly affected by eddy currents distortions. Since we are interested in smaller structures surrounding the prostate, namely the sphincters and membranous urethra, the influence of patient motion and eddy currents distortions in DTI metrics was statistically evaluated. Student’s t-test (or the Mann–Whitney test) results for comparing the mean of DTI metrics with and without motion correction have shown no statistically significant differences in the mean of each DTI metric. Additionally, the results of the ANOVA (or the Kruskal–Wallis test) for comparing the mean of DTI metrics between the three groups – no eddy currents correction, correction with FSL eddy and TOPUP and eddy currents correction option on DSI Studio – have also shown no statistically significant differences, so for the subsequent analysis no correction was considered.

### Structures architecture depicted with fiber tractography

**Fig. 7:**
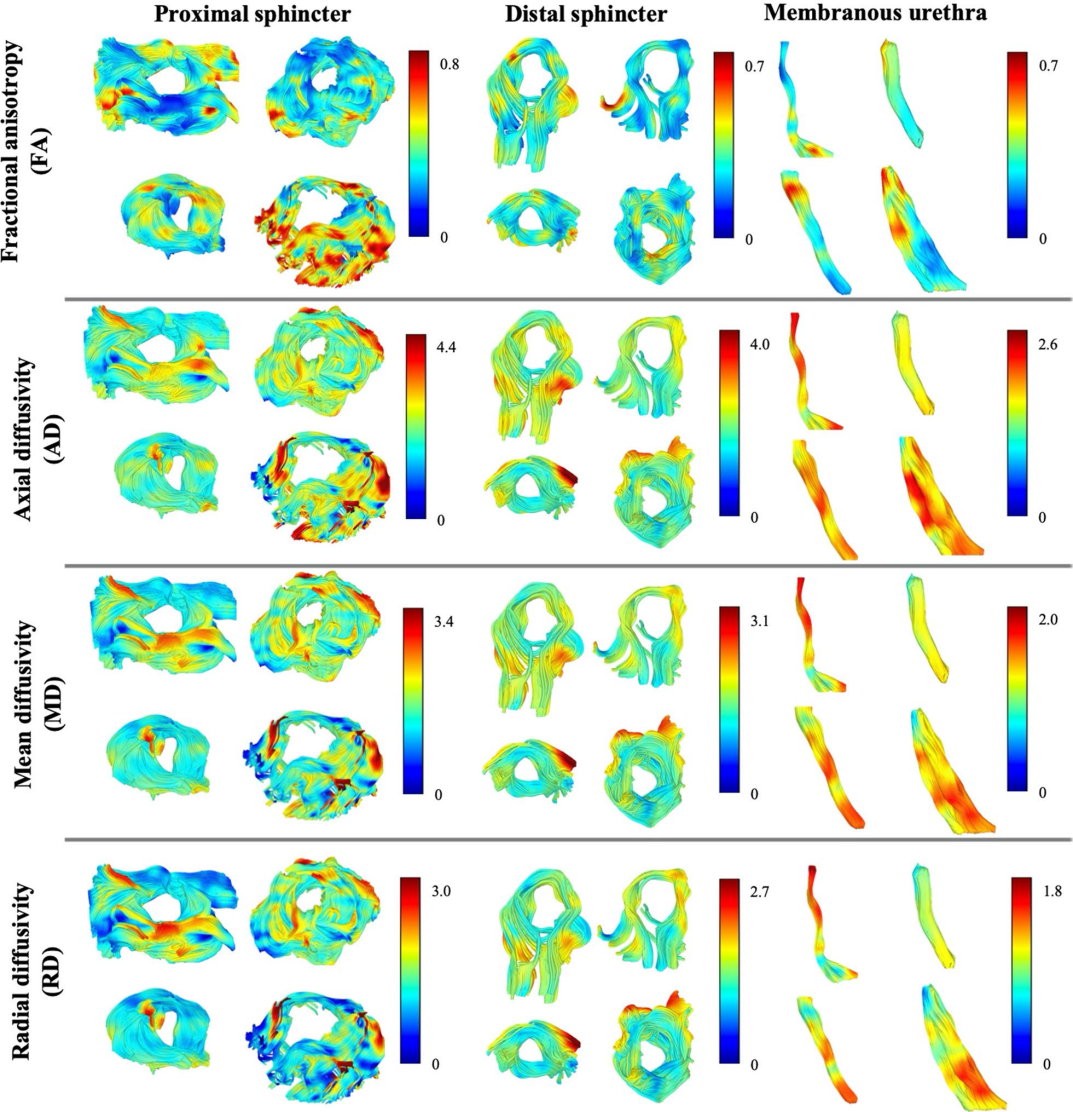
Four arbitrarily chosen representative patients’ proximal, distal sphincters and membranous urethra, color-coded using the maximum intensity of each DTI metric (from top to bottom, FA, AD, MD, and RD). Red depicts regions with a higher DTI metric value (set to the maximum value of that metric among all patients), while blue depicts regions with a DTI metric value closer to zero

For one patient, the fiber orientation of the structures of interest can be observed (Fig. 2—B1–B3) by the color-coding scheme based on the orientation of the fibers as red for left–right (*x*), green for anterior–posterior (*y*), and blue for superior-inferior (*z*). Furthermore, the fiber tractography results for the structures of interest, color-coded based on the DTI metric values, are represented on Fig. 7, for four arbitrarily chosen subjects. This allows to visualize a heterogeneity in anisotropy and diffusivity within most structures. As observed, the tracking results represent a satisfactory three-dimensional anatomical representation of muscle fibers of the male urethral sphincter complex, based on shape, size, fiber orientation, and location. Whereas the membranous urethra is a straight structure connecting the prostate apex to the penile bulb, the sphincters have circularly oriented fibers, the proximal placed between the inferior portion of the bladder and the base of the prostate with a cranio-caudal angulation, and the distal encircling the membranous urethra at the level of the prostate apex that extends until the penile bulb.

### Quantitative analysis on DTI metrics

Median, interquartile range (IQR) and range (minimum and maximum) values on each DTI metric for the proximal, distal sphincters, and membranous urethra of the patients under study are represented on Table 3. The reported values are consistent with previous studies. Regional differences in DTI metrics are shown in Tables 3 and 4, respectively, ANOVA (or Kruskal–Wallis test) results have shown that all DTI metrics were statistically significant different between analyzed structures and the corresponding post-hoc tests revealed statistically significant results on DTI metrics for most pairs of structures. Overall, results support that DTI metrics obtained can differentiate the proximal sphincter from the distal sphincter and from the membranous urethra.

**Table 3:** Median, interquartile range (IQR), and range (minimum–maximum) values on the DTI metrics of the 28 male study patients, for each analyzed structure of the urethral complex

	Proximal sphincter	Distal sphincter	Membranous urethra	*p* value
FA	0.37; 0.04 [0.31–0.46]	0.32; 0.04 [0.25–0.37]	0.33; 0.06 [0.22–0.49]	4.94e-6 *
AD	2.07; 0.18 [ 1.89–2.56]	2.08; 0.20 [1.90–2.75]	1.89; 0.22 [1.58–2.70]	6.18e-5 *
MD	1.51; 0.16 [1.37–1.91]	1.58; 0.19 [1.39–2.06]	1.41; 0.16 [1.17–1.99]	4.70e-5 *
RD	1.23; 0.15 [1.08–1.58]	1.30; 0.18 [1.13–1.71]	1.23; 0.19 [0.99–2.47]	1.12e-2 *
Tract density	1.07; 0.96 [0.23–2.62]	0.28; 0.21 [0.03–0.69]	5.57; 7.61 [0.35–52.90]	9.23e-13 *
Tract	78.6; 17.16 [5.93–118..94]	55.52; 6.55 [28.71–65.88]	26.10; 7.71 [9.82–34.8]	1.44e-15*

Tract density is expressed in tracts/mm3; Tract length in millimeters (mm); Diffusivity measures—AD, MD, and RD—in 10–3 mm2/s; FA is dimensionless. ANOVA (or the nonparametric Kruskal–Wallis test *) *p *values for the comparison of each DTI metric between regions are shown on the last column—statistically significant results are highlighted in bold

**Table 4:** Tukey’s test (or the nonparametric Dunn’s test *) *p *values for the DTI metrics comparison between each pair of regions: proximal sphincter (PROX) vs. distal sphincter (DIST); proximal sphincter (PROX) vs. membranous urethra (MU); distal sphincter (DIST) vs. membranous urethra (MU). Statistically significant results are highlighted in bold

Structure pairs	FA	AD	MD	RD	Tract density	Tract length
PROX-DIST	4.06e-6*	9.91e-1*	5.80e-2*	1.21e-2*	7.16e-6*	1.18e-4*
PROX−MU	**1.35e-3**	2.11e-4*	1.60e-2*	4.80e-l*	5.12e-3*	4.32e-16*
DIST-MU	1.31e-1*	2.11e-4*	2.60e-5*	4.51e-2*	4.97e-13*	1.55e-5*

## Discussion

The purpose of this study was to demonstrate the technical feasibility of a DTI sequence, that could potentially be implemented into the current imaging protocols to in vivo investigate the microstructure of the male urethral sphincter complex of prostate cancer patients undergoing RS-RARP. For this, various aspects on DTI of the male urethral sphincter complex were taken into account including the acquisition parameters, fiber tracking parameters and patient motion and eddy currents corrections, to optimize fiber tracking results. It was found that a single-shot DW-EPI sequence with parallel SENSE technique on a 3 T MRI scanner, using a b-value of 600 s/mm^2^ and 15 diffusion-encoding directions allowed to get a good image resolution and to minimize sequence time up to four minutes. The choice of a lower b-value comparing to that used in brain studies was made on the basis of boosting the signal to noise ratio keeping a rather comfortable acquisition time. Also, the number of diffusion encoding directions was decreased which visually did not changed the overall shape of the structures, while enabling the increase in the number of acquired slices. Additionally, the refining of fiber tracking parameters for the fiber assignment by continuous tracking (FACT) algorithm on DSI Studio yielded a good fiber tracking representation of the sphincters and the membranous urethra. In fact, the selected values on FA threshold acknowledged that lower FA caused fibers to be tracked outside the structures of interest, whereas higher values restrained too much the tracked fibers. Subject motion and eddy currents corrections of prostate diffusion tensor images were not performed in most existing studies [21–24]. In this study, it was evaluated the need for these corrections specifically for the sphincters. Since no statistically significant differences in DTI metrics were found between corrected and non-corrected datasets, patient motion and eddy currents corrections on these structures were disregarded. One probable reason for this lack of statistically significant differences is the sphincter’s small size, making it insignificantly affected by distortions. Additionally, the patients under study have taken a microlax, which should reduce the peristaltic movement and rectal air presence.

Not only the proximal and distal sphincters (circular fibers), and membranous urethra (longitudinal fibers) can be correctly depicted for twenty-eight patients, but also quantitative DTI metrics seem to be able to differentiate them. ANOVA comparisons, or the nonparametric test extension, on mean values of DTI metrics have identified statistically significant regional differences between structures, which proves the possibility of using DTI metrics to discriminate individual structures of the male urethral complex. For instance, FA in the proximal sphincter was statistically significantly increased compared with the other structures, concordantly with previous findings.

The potential utility of DTI tractography of the structures of the urethral sphincter complex has been demonstrated by Zitja et al. that have investigated pelvic floor dysfunction in female subjects [25], and by Sinha et al. that have studied its microstructure in healthy young male subjects [15]. These have been able to differentiate between the proximal and distal sphincters surrounding a central urethral bundle, and found that, in the proximal sphincter, FA was increased and tertiary eigenvalue (${\lambda }_{3}$) was decreased significantly compared with the other structures. Our intention with this work was to address a need of investigating the feasibility on prostate cancer patients, reflecting the microstructure that most accurately represents the target population that undergoes surgery. Indeed, the use of DTI to study the male urethral sphincter complex was proven feasible, not only for healthy young subjects, but also for a larger dataset of prostate cancer patients more likely to be subject to variability in shape and density due to morphologic features, tumor characteristics or advanced age might have made this technique unsustainable.

In terms of study limitations, it is important to refer this study’s relatively small sample size (28 subjects), although larger than in previous studies on the sphincters [15, 25]. In addition, the inaccessible location and the small size of the sphincters that may be corrupted by partial volume effects. Also, the ROI placement is a time-consuming manual process, that is difficult to be replaced by automatic approaches that rely on a prostate template, due to a high inter-subject anatomical variability.

In this study, the visualization and quantification of the structure of the male urethral sphincter complex before RS-RARP was demonstrated to be feasible using the proposed optimized DTI methodology. This may guide surgeons prior to the procedure or aid in a treatment informed choice by presenting to patients his rate of post-surgical continence recovery based on individual sphincteric characteristics. Further studies are needed to investigate age-related differences of the male urethral sphincter complex, to correlate DTI findings with post-surgical questionnaire test scores that measure incontinence, and eventually, to test the reproducibility of DTI as a clinical tool.

## Data Availability

Anonymized data are available upon request from the authors.

## Abbreviations

AD: Axial diffusivity
BMI: Body mass index
DW-EPI: Diffusion-weighted echo-planar imaging
FA: Fractional anisotropy
FACT: Fiber assignment by continuous tracking
IQR: Interquartile range
MD: Mean diffusivity
PA: Posterior-Anterior
PSA: Prostate-specific antigen
RD: Radial diffusivity
RS-RARP: Retzius-sparing robot-assisted laparoscopic radical prostatectomy
SENSE: Sensitivity encoding
